# Variations of Runoff and Sediment Load in the Middle and Lower Reaches of the Yangtze River, China (1950-2013)

**DOI:** 10.1371/journal.pone.0160154

**Published:** 2016-08-01

**Authors:** Na Li, Lachun Wang, Chunfen Zeng, Dong Wang, Dengfeng Liu, Xutong Wu

**Affiliations:** 1 School of Geographic and Oceanographic Sciences, Nanjing University, Nanjing, China; 2 Collaborative Innovation Center of South China Sea Studies, Key Laboratory of Surficial Geochemistry (Ministry of Education), Department of Hydrosciences, School of Earth Sciences and Engineering, State Key Laboratory of Pollution Control and Resource Reuse, Nanjing University, Nanjing, China; University of Vigo, SPAIN

## Abstract

On the basis of monthly runoff series obtained in 1950–2013 and annual sediment load measured in 1956–-2013 at five key hydrological stations in the middle and lower reaches of the Yangtze River basin, this study used the Mann-Kendall methods to identify trend and abrupt changes of runoff and sediment load in relation to human activities. The results were as follows: (1) The annual and flood season runoffs showed significant decreasing trends at Yichang station, and showed slight downward trends at Hankou and Datong stations, while the abrupt changes of dry season runoff at Yichang, Hankou and Datong stations occurred in about 2007 and the change points were followed by significant increasing trends. The construction of the Three Gorges Dam, which began to operate in 2003, influenced the variations of runoff in the mainstream of Yangtze River, but the effect weakened with the distance along the downstream direction from TGD. (2) Since the 1990s, annual sediment loads at Yichang, Hankou, and Datong stations have been decreasing significantly, and after 2002, the annual sediment load at Yichang dropped below that of Hankou and Datong. The dams and deforestation/forestation contributed to the significant decreasing trend of the sediment load. In addition, the Three Gorges Dam aggravated the downward trend and caused the erosion of the riverbed and riverbanks in the middle and lower reaches. (3) The runoff and sediment load flowing from Dongting Lake into the mainstream of the Yangtze River showed significant decreasing trends at Chenglingji station after 1970s, and in contrast, slight increase in the sediment flow from Poyang Lake to the mainstream of the Yangtze River at Hukou station were detected over the post-TGD period (2003–2013). The result of the study will be an important foundation for watershed sustainable development of the Yangtze River under the human activities.

## Introduction

Rivers play a significant role in the transport of sediment from land to the ocean affecting the evolution of the river deltas itself, which must be seen as a key pathway for material transfer on the Earth [1–2]. Additionally, river discharge is of critical importance for the lower river reaches, estuaries, and adjacent sea regions in the ecological, social and economic contexts [3–5]. In recent decades, the water discharge and sediment load of the world’s rivers have been increasingly altered by human activities and climate change [6–8]. Particularly, anthropogenic activities, such as damming, water diversion, and agriculture and domestic water consumption have significantly altered the flow and sediment regimes of rivers since the Industrial Revolution in the 18th century [9–11]. The importance of sound understanding of runoff and sediment discharge processes in a large-scale basin through different time scales is key to improve the predictions of the impact of human activities as opposed to climate change effect [12–13].

Detecting trends in river flow and sediment load data series could highlight the influences of human activities and climate changes on the river regime. A large number of researchers in the world attached great importance to the trend detection in hydrological series, such as sediment load and streamflow series at the basin scale [14–18]. This is especially important in China, where few rivers are in a natural or semi-natural condition, with disturbances causing substantial changes to runoff and sediment regimes [19].

As the largest (1,800,000 km^2^) and longest (6300 km) river in Southern Asia, the Yangtze ranks 5th globally in terms of water discharge (900 km^3^/yr) and, until recently, 4th in terms of sediment load (470 Mt/yr) [20]. With the population approaching one half billion people, the Yangtze is also one of the most heavily impacted rivers in the world [21]. Since 1950, numerous reservoirs have been constructed in the basin, with a total storage capacity of 200 km^2^ or 22% of the annual discharge of the Yangtze [22], exceeding the global average in this regard (www.seaweb.org/resources/briefings/dams.php). By 2013, 1487 large and medium reservoirs [23], among which the Three Gorges Dam is the largest, have been constructed at the Yangtze River for the purpose of irrigation, water storage for industrial and domestic uses, power generation and canalization/navigation [24].

Water discharges have been influenced by climate change, which could further impact water availability for humans [25]. However, in recent decades, intensive human activities (e.g., irrigation, dam construction, etc.) have had a major impact on the runoff and sediment load of the Yangtze River [26–29]. Zhang et al. [19] analyzed the annual runoff and annual suspended sediment loads of hydrological gauging stations along the mainstream of the Yangtze River and its main tributaries to explore the possible influences of human activities and climatic variability on the Yangtze River Basin. Xu et al. [30] used the distributed hydrological model to analyze the spatial-temporal variation of runoff in the upper Yangtze River. Yang et al. [31–32] described the response of river discharge and sediment load to anthropogenic impact and climate variability and its impacts on estuarine and coastal regions during this later period. Gao et al. [33] indicated that reservoir interception has significantly affected the fluvial sediment budget as well as the sedimentary processes of the entire Changjiang catchment basin. At the same time, sediment load and runoff variations of the Yangtze River will exert a direct influence on the accretion/recession of the Yangtze Delta [31].

Previous studies have described the water discharge and sediment load of the Yangtze River, and indicated that human activities, especially large dam construction, such as the Three Gorges Dam (TGD) in the river basin, had little influence on the annual flow regime [34]. Nevertheless, these activities had a significant impact on the sediment load of the Yangtze River [35]. However, the Three Gorges Dam began to impound water and sediment discharge on 1 June 2003 and was in full operation in 2009. It was still not sufficient to study the impact of the Three Gorges Dam on the runoff and sediment load in the middle and lower reaches of the Yangtze River, especially after the start of the full capacity operation of TGD. In addition, the measurement of the runoff and sediment load at Chenglingji and Hukou stations, which are situated at the junction of two large lakes (Dongting Lake and Poyang Lake) with the mainstream, can help to investigate the river-lake interaction (including the effects of basin discharge). Therefore, the main objectives of the study are: (a) to recognize the trend and abrupt changes of annual runoff and annual sediment load at Yichang, Hankou and Datong gauging stations on the main Yangtze River, and at Chenglingji and Hukou stations located at the outlets of Dongting and Poyang lakes; (b) to analyze the lake-river interactions and the impacts of human activities on the annual runoff and sediment load along the mainstream, especially since the construction of the Three Georges Dam; and (c) to indicate the variation of seasonal runoff at Yichang, Hankou and Datong stations, as well as to examine the anthropogenic causes.

## Data and Methodology

**Fig 1:**
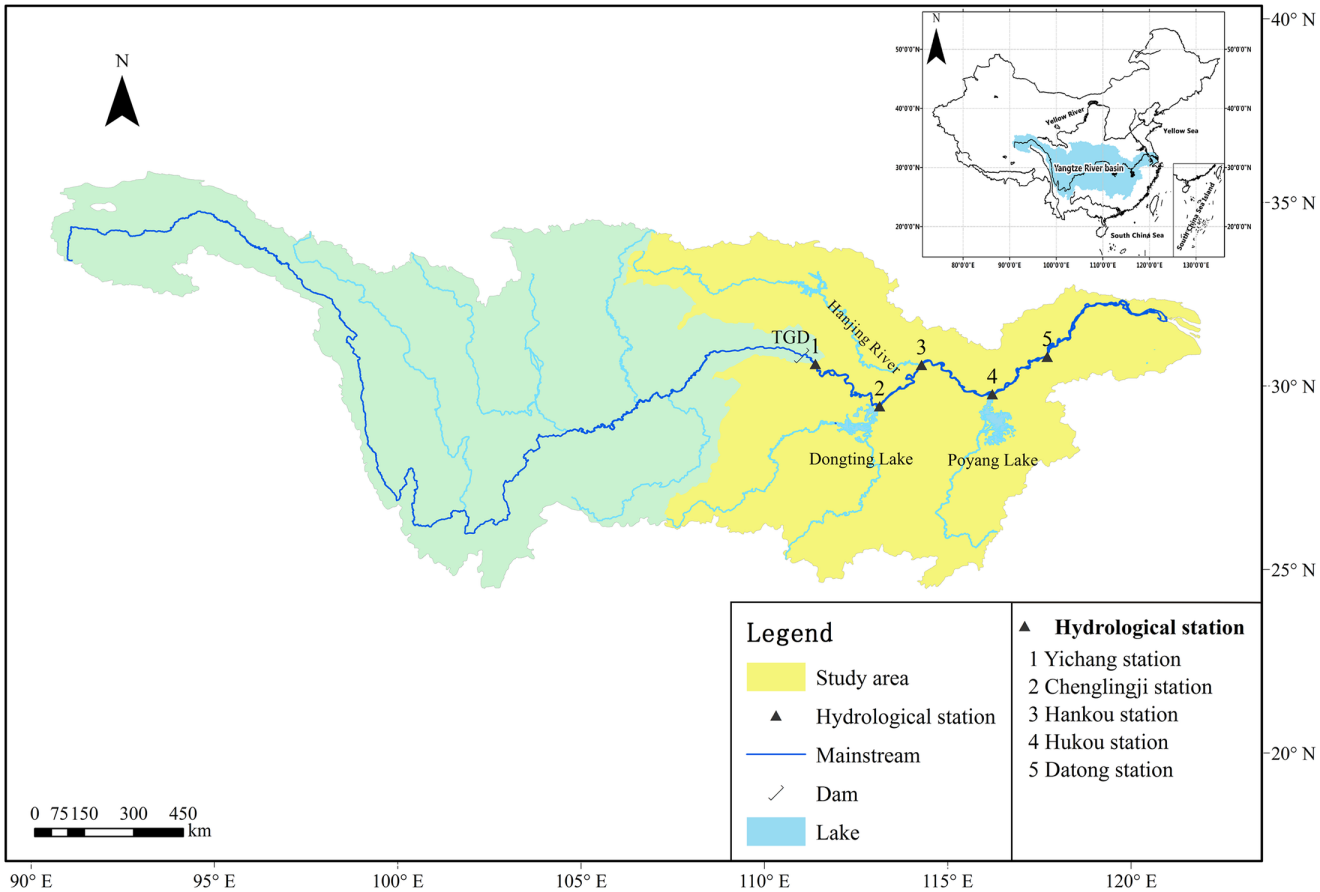
Locations of the Hydrological stations.

In the study, the mid-lower Yangtze River is considered to be located between Yichang and Datong stations, and the drainage basin covers about 700,000 km^2^ and contributes to the over 50% of the total water discharged from the Yangtze into the sea [35]. The locations and the drainage areas of the stations are presented in Fig 1. The hydrometric stations of Yichang and Datong record the water discharges from the upper basin and from the upstream into the estuary, respectively, and downstream from Datong, the river is influenced by sea-level fluctuations [36]. There are three major tributaries in the middle and down reach, i.e. Dongting and Poyang Lake drainage systems’ outflow and the Hanjiang River. Data from the Chenglingji station, which is located at the junction between Dongting Lake and the mainstream of the Yangtze River, and the Hukou station, which is located at the junction between Poyang Lake and the mainstream, are used to study the river-lake interaction.

### Data

The data on the monthly runoff and annual sediment load (SL) for the 1950–2013 period at the Yichang, Hankou and Datong hydrological stations were obtained from the Changjiang Water Resources Committee (CWRC) and the Changjiang Sediment Bulletins [37]; the annual runoff and annual sediment load (SL) data for years 1950–2013 at the Chenglingji and Hukou hydrological stations were obtained from the Nanjing Institute of Geography and Limnology. The homogeneity and reliability of the data series used in this study have been checked and found to be in good consistency. In addition to the calculations and the analysis of the annual runoff and annual sediment load at the five stations, the flood season runoff (July-September) and the dry season runoff (December-February) at the Yichang, Hankou and Datong stations were also analyzed to trace the changes in the intra-annual distribution of the runoff. The changing trends and sudden change points of the runoff and sediment load series at the five stations were detected according to the Mann-Kendall (MK) test method.

### Methods

#### Mann-Kendall test for trend

Mann [38] presented a nonparametric test for randomness against time, which constitutes a particular application of Kendall’s test for correlation commonly known as the Mann-Kendall or the Kendall test. The Mann-Kendall (MK) test is a type of nonparametric test that is widely used to detect the temporal trend for Hydrological data series [39]. Letting *X*_*t*_ = (*x*_1_, *x*_2_, …, *x*_*n*_) be a sequence of measurement, the Mann-Kendall test is

$$Z=\{\begin{array}{c} \frac{S-1}{{\left[Var(S)\right]}^{1/2}}\;if\;S>0\\ \;0\;if\;S=0\;\\ \frac{S+1}{{\left[Var(S)\right]}^{1/2}}\;if\;S<0 \end{array}$$ (1)

where

$$S=\sum_{i=1}^{n-1}\sum_{j=i+1}^{n}\text{sgn}\left(x_{j}-x_{i}\right)$$ (2)

$$\text{sgn }\left(x\right)=\{\begin{array}{c} 1\text{ if }x>\text{0}\\ 0\text{ if }x=\text{0}\\ -1\text{ if }x<\text{0} \end{array}$$ (3)

E(S)=0 (4)

$$Var\left(S\right)=\frac{n\left(n-1\right)\left(2n+5\right)}{18}$$ (5)

Where *n* is the data length and *Var*(*S*) is the variance of *S*. The positive value indicates that there is an upward trend in which the observations increase with time, and on the other hand, a negative value of *Z* means that there is a downward trend [40]. In a two sided test for a trend, if |*Z*|>*Z*_*α*/2_, where *Z* is asymptotically normally distributed and *Z*_*α*/2_ is the critical value of the standard normal distribution with a probability *α*/2, the trend of the sequence will be significant [41].

#### Mann-Kendall test for abrupt change

The Mann-Kendall test for abrupt change follows Gerstengarbe and Werner [42] who used the method to test an assumption about the beginning of the development of trend within a sample (*x*_1_, *x*_2_, …, *x*_*n*_) of the random variable *X*, based on the rank series of the progressive and retrograde rows of this sample. The MK test statistic is given by

$$UF_{k}=\frac{s_{k}-E(s_{k})}{\sqrt{Var(s_{k})}}\;k=1,2,\cdots ,n$$ (6)

where

$$s_{k}=\sum_{i=1}^{k}r_{i}\;r_{i}\text{ = }\{\begin{array}{c} 1\;x_{i}>x_{j}\\ 0\text{ else } \end{array}\;j=1,2,\cdots ,n$$ (7)

$$E(s_{k})=\frac{n(n+1)}{4}$$ (8)

$$\;Var(s_{k})=\frac{n(n-1)(2n+5)}{72}$$ (9)

Where, under the null hypothesis of no trend, the statistic *s*_*k*_ is distributed as a normal distribution with the expected value of *E*(*s*_*k*_) and the variance *Var*(*s*_*k*_). Following the same procedure as shown in Eqs (5) ~ (8), the variables, *s*_*k*_, *E*(*s*_*k*_) and *Var*(*s*_*k*_), will be calculated for the retrograde sample, and the result value, *UB*_*k*_, will be the retrograde series of *UF*_*k*_. Under the above assumption, *UF*_*k*_ follows the standard normal distribution. In a two-sided test for a trend, the null hypothesis is rejected at the significance level of *α*, if |*UF*_*k*_|>*UF*_(1−*α*/2)_, where *UF*_(1−*α*/2)_ is the critical value of the standard normal distribution with a probability exceeding *α*/2.

In this paper, the significant level of α = 5% is used. The intersection point of the two lines, *UF*_*k*_ and *UB*_*k*_, indicates the point in time of the beginning of a developing trend within the time series. The null hypothesis (the sample is not affected by a trend) must be rejected if the intersection point is significant at 5% significance level (i.e. outside the 95% confidence interval).

## Results

### Alterations in annual runoff and annual sediment load

According to the statistical data on the annual runoff and annual sediment load at the five hydrological stations in the middle and lower Yangtze River between 1950–2013 (S1 and S2 Figs), the average annual runoff at the Yichang, Chenglingji, Hankou, Hukou and Datong stations was 4.30×10^11^m^3^, 2.86×10^11^m^3^, 7.07×10^11^m^3^, 1.5×10^11^m^3^ and 8.91×10^11^m^3^, respectively. The multi-year average of the sediment load at Yichang, Hankou and Datong stations was 4.01×10^8^ tons, 3.45×10^8^ tons and 3.67×10^8^ tons, respectively, and the average of the sediment load at Chenglingji and Hukou was 3.5×10^7^ tons and 9.87×10^7^ tons. During the period of 1950–2013, the maximum sediment load at Yichang, Hankou and Datong was 7.54×10^8^ tons, 6.15×10^8^ tons and 6.78×10^8^ tons, respectively, while the sediment load at Yichang, Hankou, Datong was 0.3×10^8^ tons, 0.928×10^8^ tons and 1.17×10^8^ tons, respectively, in 2013.

Table 1 shows the Mann-Kendal test values for the trend changes of the annual runoff and annual sediment load at the five hydrometric stations in the middle and lower Yangtze River. The Mann-Kendall test for the trend indicated that the annual runoff at Yichang and Chenglingji showed significantly decreasing trends (P<0.05), whereas the annual runoff at Hankou and Datong demonstrated gently decreasing trends (P>0.05). On the other hand, there was a gently increasing trend in the annual runoff at Hukou in 1950–2013. In addition, the annual sediment load at Yichang, Chenglingji, Hukou and Datong showed significantly decreasing trends (P<0.0001) except at the Hukou. There were significant inter-annual variations of sediment load in the mainstream of Yangtze River.

**Table 1:** MK test for the runoff and sediment load series at the five hydrometric stations.

	Yichang		Chenglingji		Hankou		Hukou		Datong	
	Z^a^	P^b^	Z	P	Z	P	Z	P	Z	P
Annual runoff	-2.144	<0.05	-4.612	<0.05	-0.742	>0.05	0.237	>0.05	-0.666	>0.05
Annual sediment load	-5.829	<0.0001	-8.425	<0.0001	-6.789	<0.0001	-0.416	>0.05	-7.037	<0.0001

^a^ if Z>0(<0), MK test indicates an increasing (decreasing) trend;

^b^ if P<0.05(>0.05), the trend of runoff series indicates an significance (no significance) changes;

**Fig 2:**
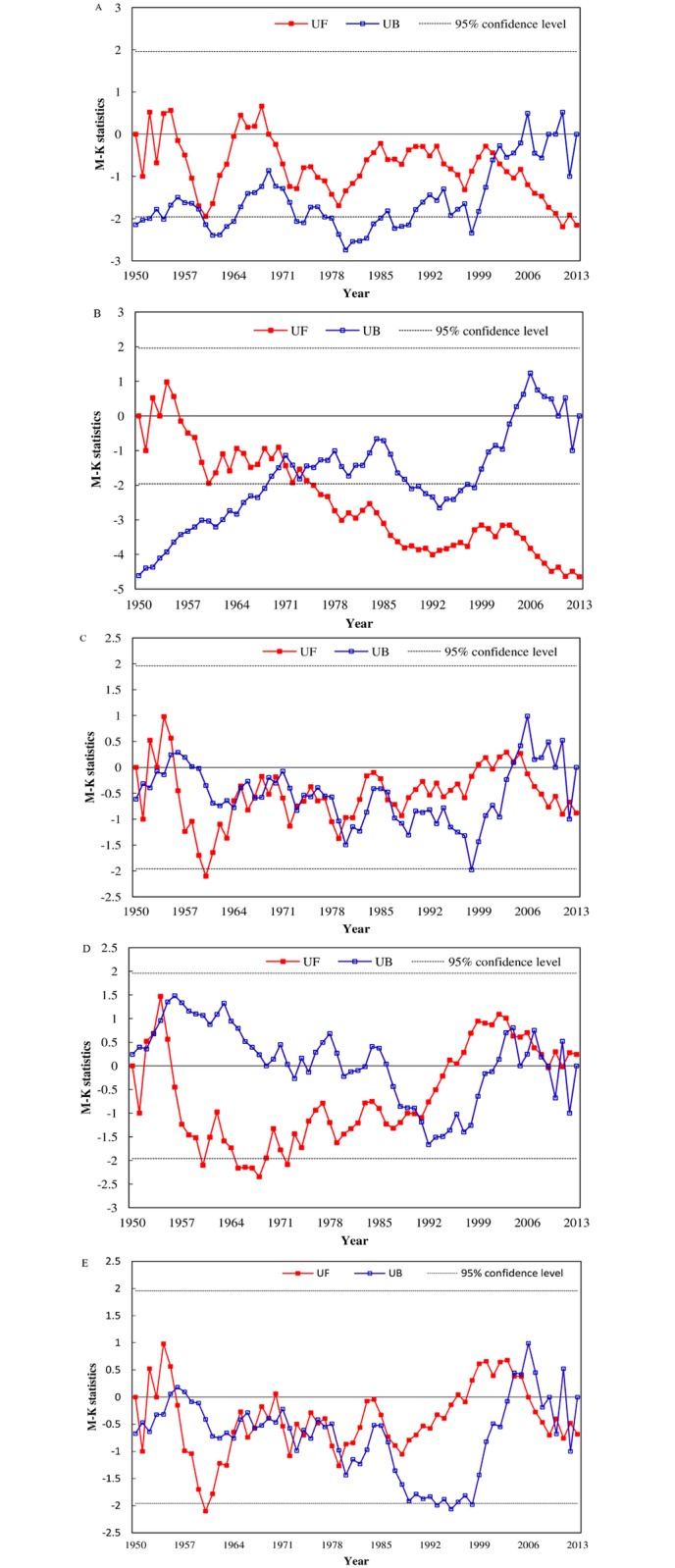
Mann-Kendall values of the annual runoff at the following stations: (A) Yichang, (B) Chenglingji, (C) Hankou, (D) Hukou and (E) Datong.

The Mann-Kendall values of the annual runoff from 1950 to 2013 at Yichang, Chenglingji, Hankou, Hukou and Datong is shown in Fig 2. The change trend of the annual runoff at the five stations was similar to the analysis in Table 1. The sudden changes and the changing patterns at the five stations were different in past decades.

Before 1960, the annual runoff of the five stations experienced almost the same fluctuant trend. There was no abrupt change in the annual runoff at Yichang during the period of 1961–2000 (Fig 2A). The sudden change point (intersection point of UF and UB line) of the annual runoff at Yichang was in 2001, and the annual runoff became a significant decreasing trend after 2011 (>95%confidence level). The Mann-Kendall analysis of the annual runoff at Chenglingji showed that the annual runoff has had a significant decline trend (more than 95% confidence level) since 1970, which was the abrupt change point (Fig 2B). From 1961 to 1990, the average annual runoff at Hukou showed a declining trend, and in 1960, 1964–1969 and 1974 the annual runoff had a significant decreasing trend. However, after 1995, the trend for the annual runoff at Hukou was gently increasing (Fig 2D).

During 1961–2013, the fluctuation trends of the annual runoff at Hankou and Datong were similar (Fig 2C and 2E). The MK values of the annual runoff at the two hydrological stations were within the critical values at the significance level of *α* = 5%. There was no obvious abrupt change in the annual runoff at Hankou and Datong, but after 2005, the trend was mildly decreasing.

**Fig 3:**
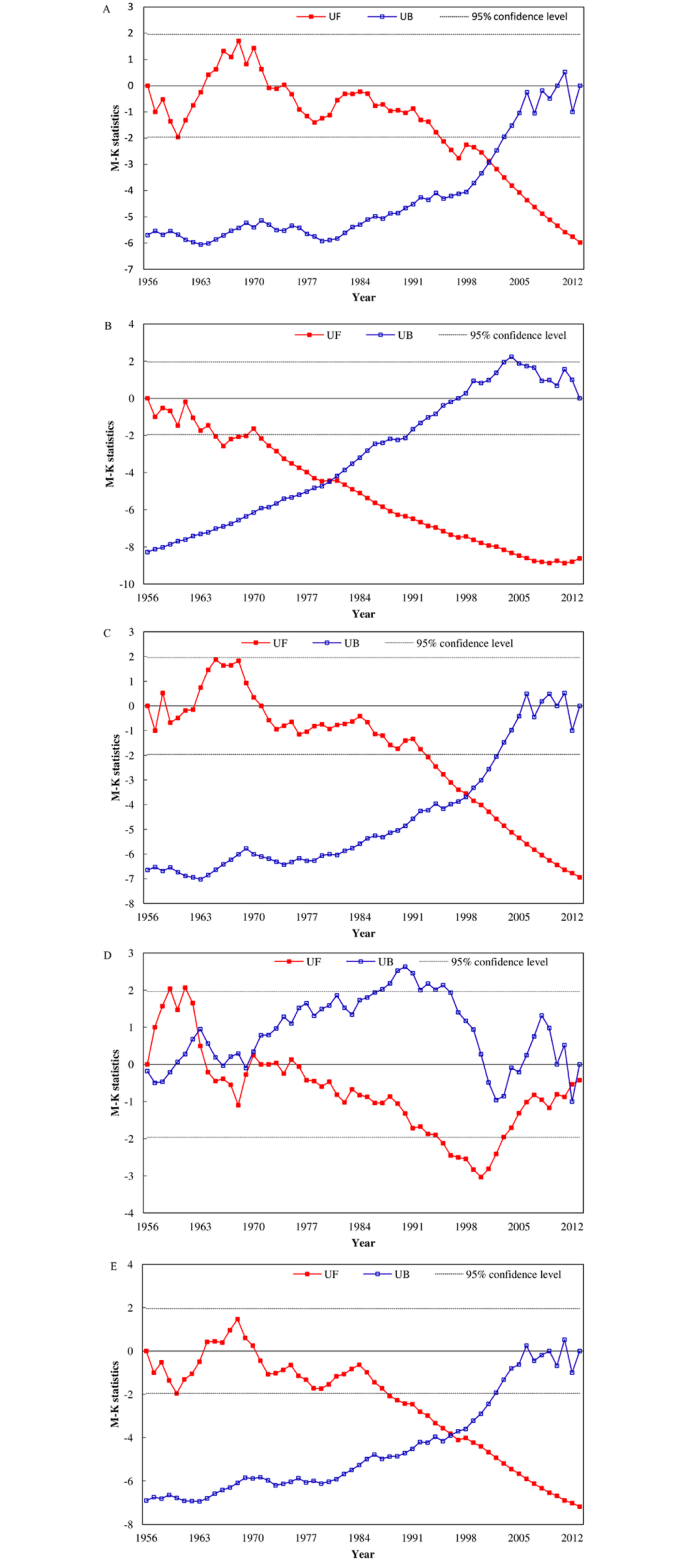
Mann-Kendall values of the annual sediment load at the following stations: (A) Yichang, (B) Chenglingji, (C) Hankou, (D) Hukou and (E) Datong.

The Mann-Kendall values of the annual sediment load at Yichang, Chenglingji, Hankou, Hukou and Datong were presented from 1956 to 2013, as shown in Fig 3. The annual sediment load at Yichang, Chenglingji, Hankou, and Datong showed a significant declining trend (> 95% confidence level), and the trend for the annual sediment load of Hukou was gently declining.

From 1956 to 2013, the Mann-Kendall values of the sediment load at Yichang, Hankou and Datong were very similar in their fluctuant trend (Fig 3A, 3C and 3E). In 1960s, the sediment load at Yichang, Hankou and Datong showed a gentle increase, not significant at a 95% confidence level. From 1995, the annual sediment load at Yichang showed a significant declining trend (>95% confidence level), and the abrupt change occurred in 2001, after which the decreasing trend intensified. Similarly, the annual sediment load at Hankou showed a significant declining trend (>95% confidence level) after 1993 and the trend for the annual sediment load at Datong was significant declining (>95% confidence level) after 1988. The abrupt change in the annual sediment load at Hankou and Datong occurred in 1998 and 1996, respectively, after which the decreasing trends intensified.

After 1965, the annual sediment load at Chenglingji showed a significant declining trend (>95% confidence level), and the abrupt change occurred in 1980, after which the decreasing trend of the annual sediment load intensified (Fig 3B). The annual sediment load at Hukou was in a slightly increasing trend during 1956–1963, and after 1970, the annual sediment load showed a declining trend, especially during the period of 1995–2002 (Fig 3D), when the trend was significantly decreasing (> 95% confidence level).

### Alterations in season runoff

In addition to the results of the annual runoff trend analysis, the season runoff of Yichang, Hankou, and Datong stations were chosen to detect the intra-annual changes. The average flood season runoff at Yichang, Hankou, and Datong stations was 2.15×10^11^m^3^, 3.0×10^11^m^3^ and 3.53×10^11^m^3^, respectively, and the average dry season runoff was 0.37×10^11^m^3^, 0.73×10^11^m^3^ and 0.97×10^11^m^3^, respectively (S3 Fig). The average of flood season runoff at Yichang, Hankou and Datong was three to five times higher than the average of the dry season runoff for 1956–2013 period. There were obvious seasonal fluctuation of the runoff in the middle and lower reaches of the Yangtze River.

**Fig 4:**
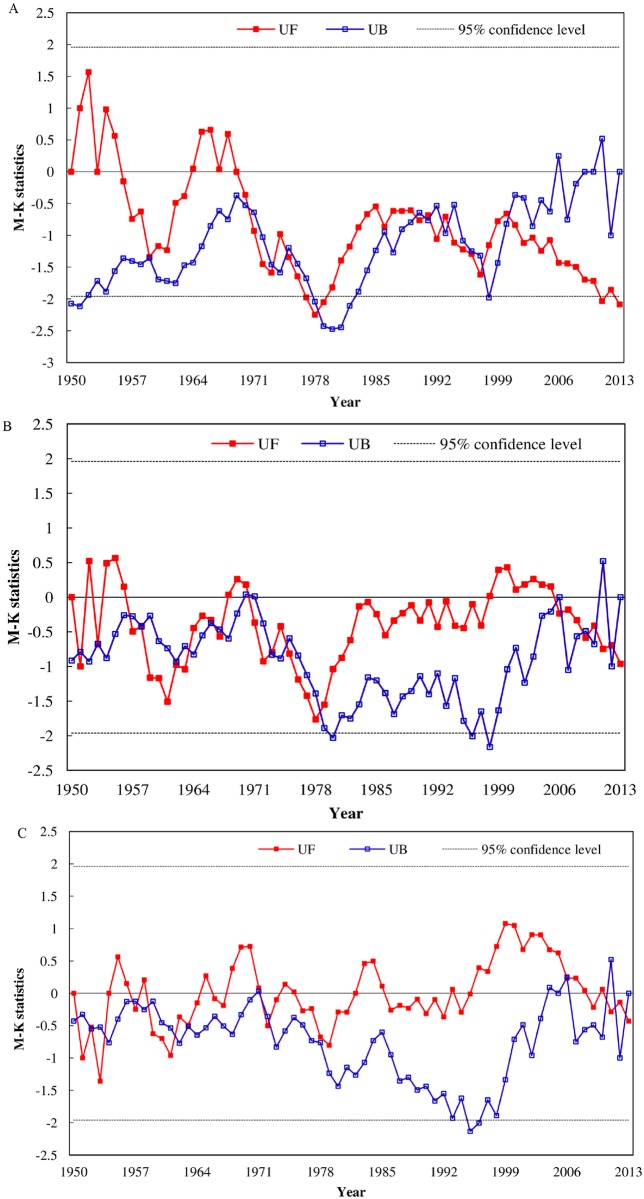
Mann-Kendall values of the flood season runoff at the following stations: (A) Yichang, (B) Hankou and (C) Datong.

**Fig 5:**
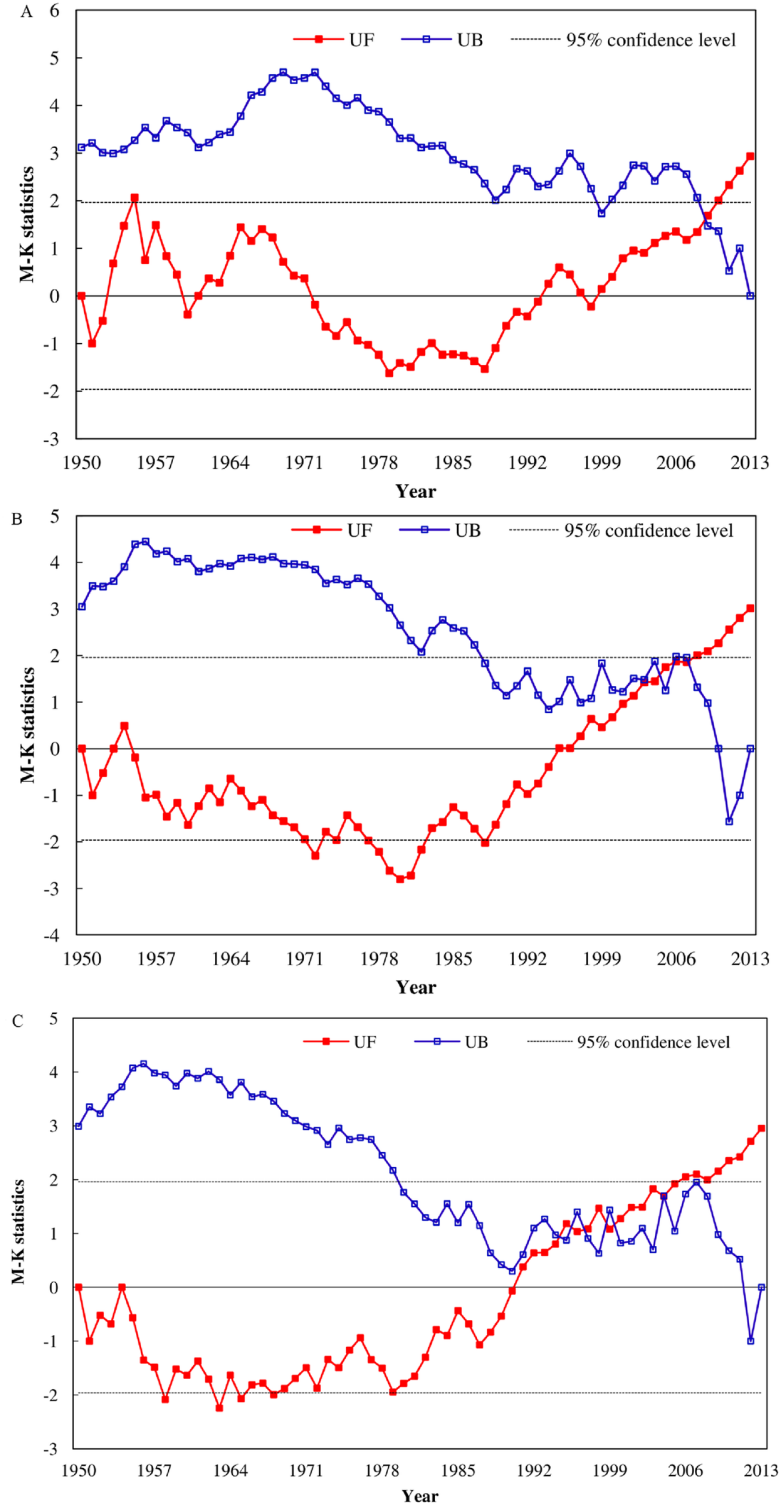
Mann-Kendall values of the dry season runoff at the following stations: (A) Yichang, (B) Hankou and (C) Datong.

The flood season runoff at Yichang showed significant decreasing trends (P<0.05), whereas the dry season runoff at Yichang, Hankou and Datong had significant increasing trends (P<0.05), as shown in Table 2. The Mann-Kendall values of the flood season runoff and dry season runoff at Yichang, Hankou and Datong during 1950–2013 were shown in Figs 4 and 5.

**Table 2:** MK test for the season runoff series at the three hydrometric stations.

	Yichang		Hankou		Datong	
	Z	P	Z	P	Z	P
Flood season	-2.074	<0.05	-0.933	>0.05	-0.423	>0.05
Dry season	3.018	<0.05	3.036	<0.05	2.746	<0.05

The abrupt change of the flood season runoff at Yichang occurred in 2000, and the flood season of Yichang became to be significantly decreasing after 2011. By contrast, there was no obvious abrupt change in the flood season runoff at Hankou and Datong.

As shown in Fig 5, the sudden change points of the dry season runoff at Yichang, Hankou and Datong occurred in 2009, 2007 and 2007, respectively. After the abrupt change year, the trend for the dry season runoff at the three stations was significantly increasing (>95% confidence level).

## Discussion

Anthropogenic influences and changing climate can affect the “normal” supply and flux of runoff and sediment along hydrological pathways [10, 43]. Until now, the statistic data of International Commission on Large Dams (ICOLD) suggested there has been >58000 dams no less than 15 meters, capable of holding back >15000 km^2^ of water, and China had the most dams in the world. The construction of dams for water supply, irrigation and flood control represent a key element of water resource exploitation in many areas of the world and sedimentation behind such dams must result in a substantial decrease in the downstream sediment flux, as shown in Table 3. In the large rivers, the catchment-scale impact of dams on the changes of water and sediment load were well recognized and the reservoir construction was probably the most important influence on land-ocean sediment fluxes [16].

**Table 3:** Comparison of studying effects of dams on water and sediment in large rivers.

References	Rivers	Subjects	Influence Factor
Fanos [44]; Weigel [45]	Nile River	Water; Sediment loads	Dams
Abam [46]	Niger River	Water; Sediment loads	Dams
Mikhailova [47]; Batalla et al. [48]	Ebro River	Sediment loads	Dams
Topping et al. [49]; Carriquiry et al. [50]; Zamora et al. [51]	Colorado River	Water; Sediment loads	Dams
Sivakumar and Wallender [52]; Blum and Roberts [53]	Mississipi River	Sediment loads	Dams
Malini and Rao [54]; Rao et al. [55]	Godavari and Krishna River	Sediment loads	Dams
Yang et al. [56]; Xu [57,58]; Yang and Saito [59]; Chu et al. [60]; Ran et al. [61]	Yellow River	Water; Sediment loads	Human activities (dams and water consumption); Climate changes
Dai et al. [62]; Chen et al. [63]; Zhang et al. [17]	Pearl River	Water; Sediment loads	Dams; Climate changes

Like the above large rivers, the variations of runoff and sediment load in Yangtze River Basin have been drawn more attentions, due to the reason that changes could reflect the human activities versus climate changes [64]. The mean annual precipitation (MAP) in the Yangtze basin over the period of 1955–2011 was 1045 mm, with a range from 1207 to 908 [65], and the annual precipitation in Yangtze basin in 2013 was 1029 mm [23]. There has been no evidence of a decrease in the annual precipitation series or an increase in the annual evapotranspiration series. On the contrary, 47,842 reservoirs were constructed in the Yangtze River Basin until 2012. And the Gezhouba Dam and the Three Gorges Dam were constructed in the mainstream of the Yangtze River [66]. In the mainstream of Yangtze River, the Yichang station is the control point of the upper Yangtze River Basin and is located at the begin of the middle reach of the Yangtze River, 44-km downstream from the Three Gorges Dam [34]. And the Datong station, downstream from the Yichang and Hankou stations, records the water and sediment flux from Yangtze River to the estuary. Therefore, the study will pay more attention on the trend variations of flow and sediment regimes caused by the human activities, especially the TGD.

**Fig 6:**
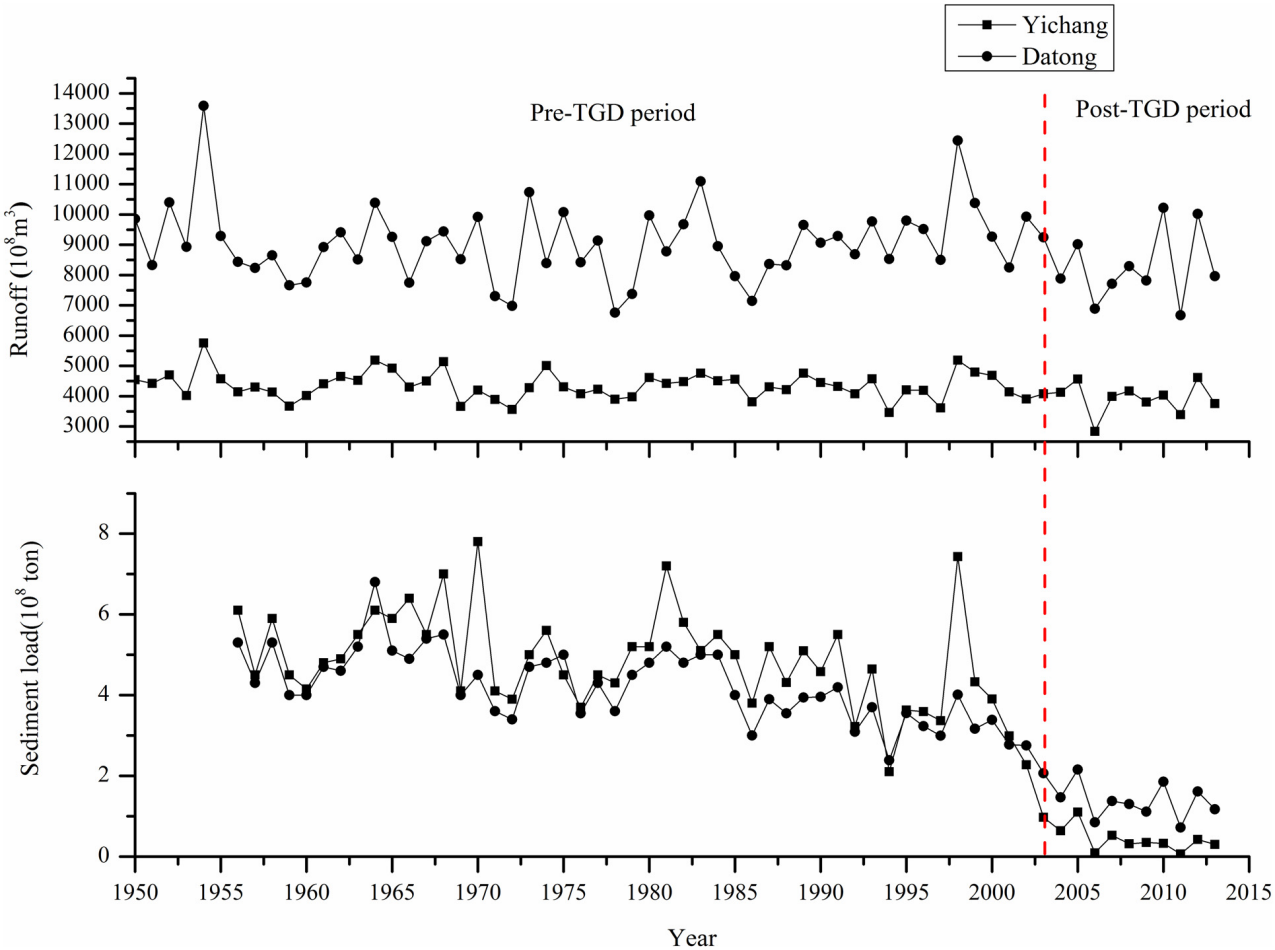
The temporal variations of the annual runoff and sediment load at Yichang and Datong.

The annual and season runoff at Yichang, Hankou and Datong were analyzed to educe the intra-annual, inter-annual and space-time change features of the water regime in the mainstream of the Yangtze River. Compared with Hankou and Datong, the annual runoff and flood season (July-September) runoff at Yichang showed a significant decreasing trend, both of which abrupt change occurred in 2001 (Figs 2A and 4A). The abrupt change of dry season (December-February) runoff at Yichang, Hankou and Datong occurred around 2007, after which there was a significant increasing trend (> 95% confidence level) (Fig 5). The Three Gorge Dam, which has seasonally regulated its water storage, has began to store in autumn and drain it in January next year since 2003. Between the pre-TGD period (1950–2001) and post-TGD periods (2003–2013), the average multi-annual runoff at Yichang reduced 417×10^8^m^3^ (9.6%) and the runoff flow into the sea at Datong decreased 735×10^8^m^3^ (8.1%) (Fig 6), and on the contrary, the dry season runoff at Yichang and Datong increased 54×10^8^m^3^ (15.1%) and 94×10^8^m^3^ (9.76%), respectively (S3 Fig). After the construction of the TGD, variation trends of hydrological regimes at Yichang station were more obvious than those at Hukou and Datong stations, and the TGD was the main reason to cause the changes of the dry season runoff in the mainstream of the Yangtze River.

The annual sediment load at Yichang, Hankou and Datong had a significant declining trend (>95% confidence level) during the period of 1956–2013 (Table 1), and the abrupt changes occurred in 1990s due to the reservoirs and soil and water conservation project in the upper reaches of the Yangtze River [19,28,67]. Over the post-TGD period (2003–2013), the degree of reduction in the annual sediment load at Yichang and Datong increased, at which the average multi-annual sediment load decreased by 90.4% and 66%, respectively, compared with the pre-TGD period (Fig 6). Meanwhile, the annual sediment load at Yichang was less than at Datong from 2002 (Fig 6), and therefore the erosion would occur in the middle and lower reaches of the Yangtze River mainstream.

Besides the middle and lower reaches of the Yangtze River, the changes of hydrologic regime at Chenglingji and Hukou stations at the junctions of the Dongting Lake and Poyang Lake with the Yangtze River could understanding the river-lake interactions. Combined with the existing researches, the study indicated the causes of variation of water and sediment regimes from two tributaries to Yangtze River. The abrupt changes of the annual runoff and annual sediment load at Chenglingji occurred in 1970 (Figs 2B and 3B), which were followed by significant declining trends (> 95% confidence level). The average multi-annual runoff and average multi-annual sediment load at Chenglingji decreased by 16.53% and 40.6% between 1970–2002 and 3003–2013, respectively (S1 and S2 Figs). Under the combined influence of water and sediment reduction from the three outlets in the Jingjiang River, the storage function of the Dongting Lake and the reduction of the sediment load from the four rivers [68, 69], the runoff and sediment load outflow from Dongting Lake towards the main river at Chenglingji showed a significant decreasing trend, and the Three Gorges Dam intensified impacts on the trend. On the other side, the annual sediment load at Hukou showed a significant decline during mid-1990s (>95% confidence level) (Fig 5D), due to the facts of the total reservoir capacity was 2.363×10^9^ m^3^ in the upper reaches of the Ganjiang River until 1983 and the Wan'an Reservoir was closed in 1990. Although the sediment load of the combined five rivers reduced, due to sand mining the sediment concentration of Poyang Lake increased after 2000 and the average annual sediment load at Hukou increased significantly [70,71]. As in Fig 3D, the amount of sediments transported from Poyang Lake into the mainstream of the Yangtze River started to increase after 2000. Gao et al. [72] suggested that the average frequency of the backflow of the Changjiang River as well as the quantity of water and sediment backflow lowered in 2003–2010, especially after the sediment transport of the main river further decreased after the impoundment of the TGD in 2003. Therefore, the TGD has an indirect impact on the water discharge and sediment flux changes between two large lake tributaries and the Yangtze River.

## Conclusions

Similar to the large rivers in Table 3, the human activities, such as dam construction, deforestation/forestation, and sand mining, exerted an increasing impact on the water discharge and sediment flux. Moreover, as the largest dam in the mainstream, the impact of the Three Gorges Dam on the changes of the runoff and sediment load in the middle and lower reaches of the Yangtze River was discussed inevitably. The Three Gorge Dam intensified the decreasing trend of the annual runoff and annual sediment load in the mainstream, and the impact decreased with the increasing distance between the Three Gorges Dam and the hydrological stations. Over post-TGD period (2003–2013), the dry season runoff along the mainstream of the Yangtze River had a significant increasing trend (>95% confidence level). Moreover, after the impoundment of the TGD, the runoff and sediment load outflow from Dongting Lake towards the main river at Chenglingji decreased, and in addition, the average frequency of the backflow of the Yangtze River as well as the quantity of water and sediment backflow lowered and the amount of sediments transported from Poyang Lake into mainstream started to increase.

The research indicated that the construction of the Three Gorges Dam project had a direct and indirect effect on the variations of hydrological regime in the middle and lower reaches of the Yangtze River. The Middle Route and East Route of the South-to-North Water Diversion have began to transport water in 2014, and the water reservoirs and water diversion projects in the watershed would continue in coming decades, which would continue to reduce the sediment load and water discharge and result in the erosion in the main river channel. For the sustainability of water resources utilization, the water reservoirs and water diversion project in the watershed should consider the impact in the hydrologic regime in the middle and lower reaches of the Yangtze River.

## Supporting Information

S1 Fig Time series of annual runoff at Yichang, Hankou, Datong, Chenglingji and Hukou stations. (TIF)

S2 Fig Time series of annual sediment load at Yichang,Hankou,Datong, Chenglingji andHukou stations. (TIF)

S3 Fig Time series of season discharge at Yichang, Hankou and Datong stations. (TIF)
