# Supplementary figures and images for: Variations of Runoff and Sediment Load in the Middle and Lower Reaches of the Yangtze River, China (1950-2013)
Source: PLoS One. 2016 Aug 1;11(8):e0160154. doi: 10.1371/journal.pone.0160154 (PMC4968824; doi:10.1371/journal.pone.0160154)

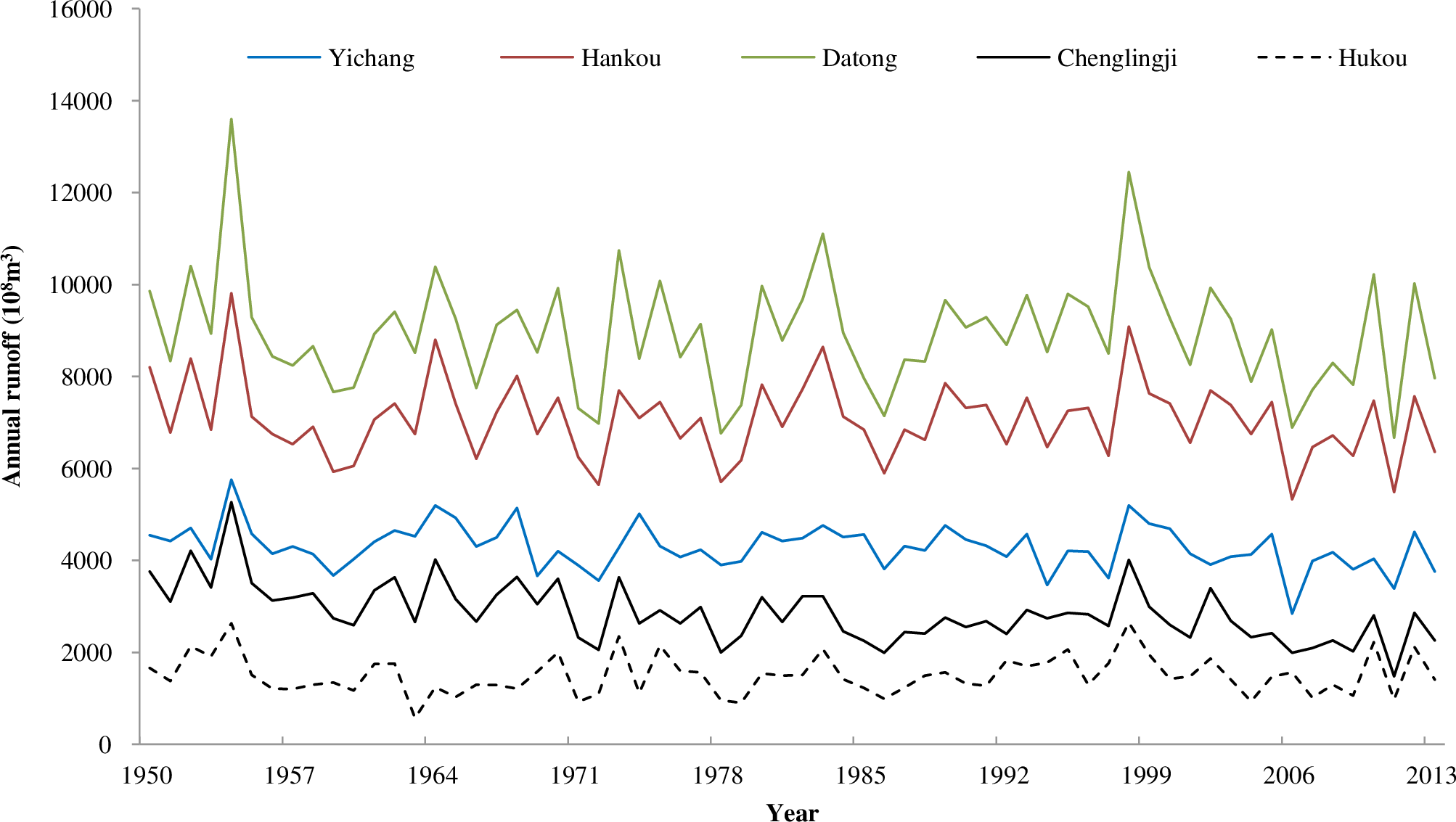

Supplement: S1 Fig — (TIF) [file pone.0160154.s001.tif]

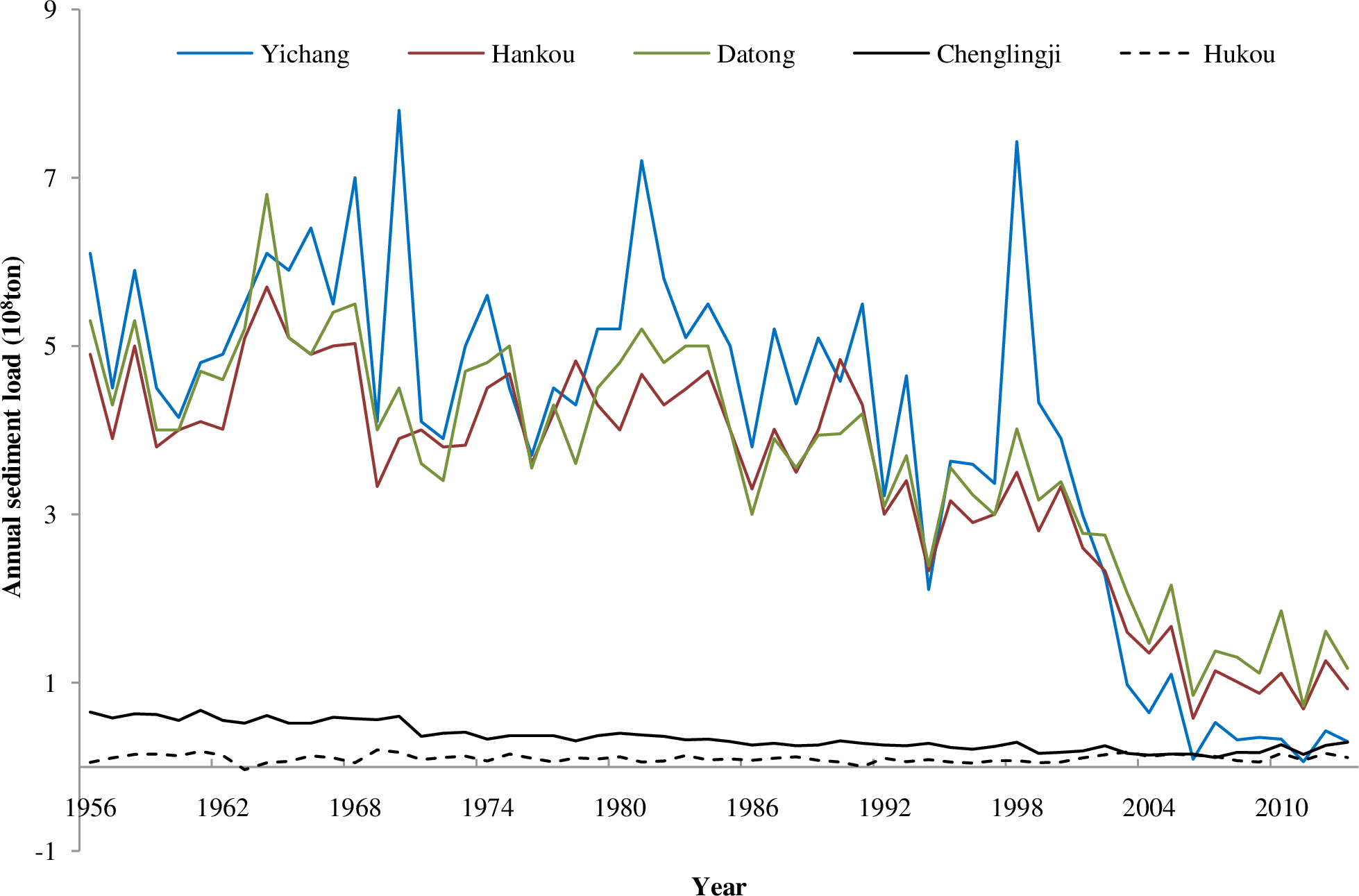

Supplement: S2 Fig — (TIF) [file pone.0160154.s002.tif]

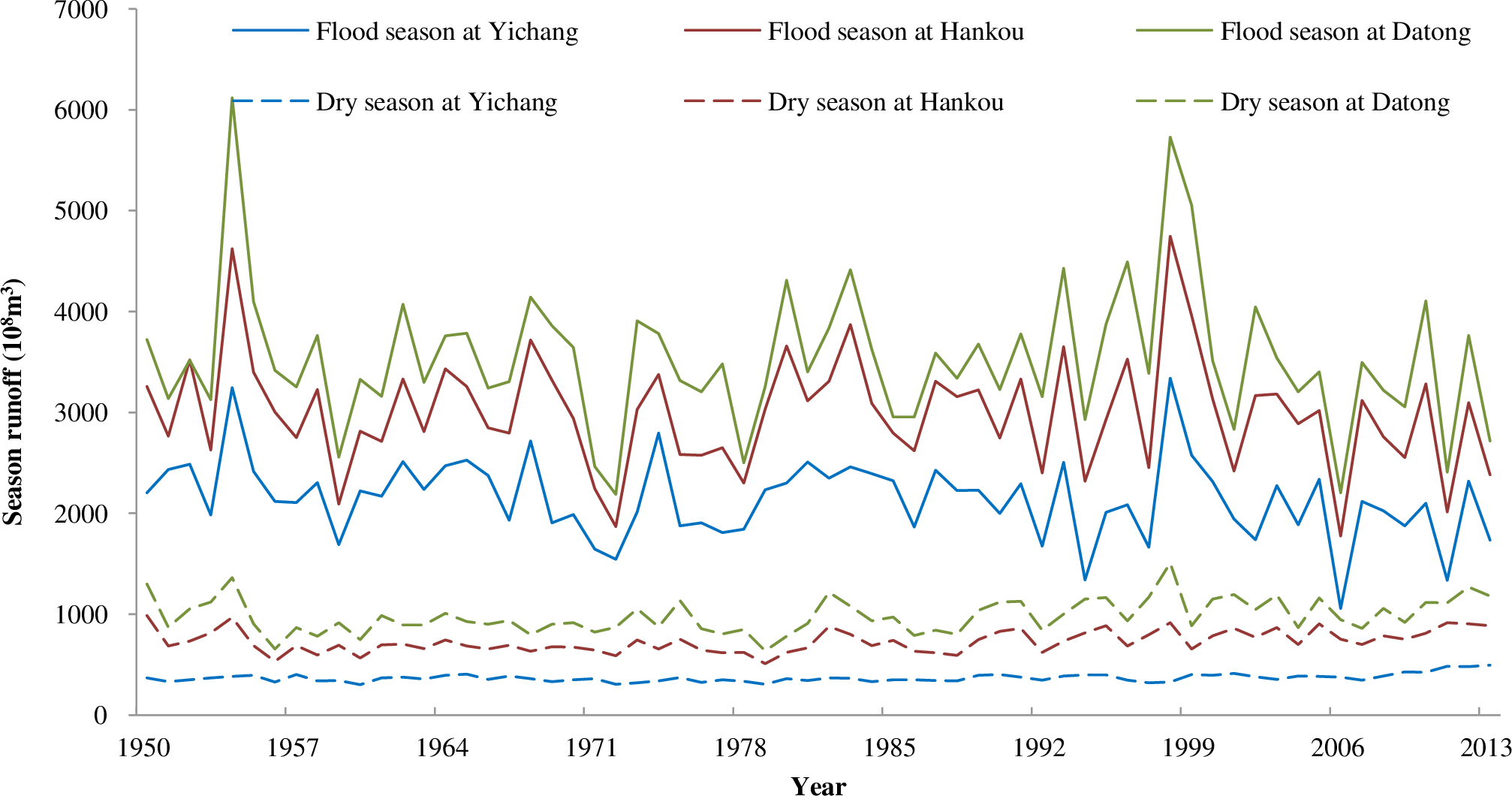

Supplement: S3 Fig — (TIF) [file pone.0160154.s003.tif]
